# Enhancing Antioxidant Activity and Nutritional Profile of Dark Chocolate Through Enrichment with Plant Sterols: A Study on Phytosterol Concentrations and Functional Properties

**DOI:** 10.3390/foods13223578

**Published:** 2024-11-09

**Authors:** Patrycja Topka, Magdalena Rudzińska, Szymon Poliński, Aleksandra Szydłowska-Czerniak, Małgorzata Tańska

**Affiliations:** 1 Department of Food Plant Chemistry and Processing, Faculty of Food Sciences, University of Warmia and Mazury in Olsztyn, 10-718 Olsztyn, Poland; patrycja.topka@uwm.edu.pl; 2 Department of Food Technology of Plant Origin, Poznań University of Life Sciences, Wojska Polskiego 28, 60-637 Poznań, Poland; magdalena.rudzinska@up.poznan.pl; 3 Department of Analytical Chemistry and Applied Spectroscopy, Faculty of Chemistry, Nicolaus Copernicus University in Toruń, Gagarina 7, 87-100 Toruń, Poland; szymon.polinski@wp.pl

**Keywords:** conching, tempering, antioxidant capacity, total phenolic content, sterols, stanols

## Abstract

Chocolate, particularly dark chocolate, is recognized for its antioxidant properties attributed to the presence of flavonoids that promote cardiovascular health. Enriching chocolate with phytosterols, naturally occurring plant compounds known to be effective in reducing cholesterol levels, has the potential to enhance cardiovascular benefits. The incorporation of phytosterols into chocolate provides a palatable and cost-effective means of delivering these beneficial compounds to the body. This study examined the concentrations of sterols and stanols, as well as the antioxidant properties of dark chocolate enriched with plant-derived sterols and stanols. A commercially available preparation containing phytosterol esters (Vegapure^®^ 95 WE) was utilized for this enrichment. Four levels of phytosterol esters (3, 6, 9, and 12%) were added at two distinct stages of chocolate processing: conching and tempering. Sterol and stanol contents were analyzed chromatographically, total phenolic content was determined using the Folin–Ciocalteu method, and antioxidant capacity was assessed via the DPPH assay. Additionally, a sensory evaluation was performed to assess the palatability of the enriched chocolates. The enriched chocolates showed significantly increased levels of sitosterol (up to 1117.68 mg/100 g), campesterol (up to 119.10 mg/100 g), and sitostanol (up to 76.42 mg/100 g). The antioxidant capacity of the enriched dark chocolates was more strongly correlated with phenolic compound content than with phytosterol content. Sensory differences, particularly in taste, were also noted, influenced by the addition of phytosterols. The stage at which phytosterol esters were introduced affected the chocolate’s properties, with the tempering stage proving to be the more advantageous step for incorporating phytosterols, resulting in a lower loss of bioactive compounds. These findings suggest that enriching dark chocolate with phytosterols improves its nutritional profile and functional properties, positioning it as a potential dietary supplement for cholesterol management and cardiovascular health.

## 1. Introduction

The development of functional foods has emerged as a key strategy in the prevention and management of chronic diseases, particularly those related to cardiovascular health [1]. Among these functional foods, dark chocolate has attracted significant attention due to its high content of bioactive compounds, such as flavonoids, which are well known for their strong antioxidant properties [2]. These compounds have been extensively researched for their ability to reduce oxidative stress, decrease inflammation, and enhance endothelial function, all of which contribute to improved cardiovascular health [3]. However, the potential of dark chocolate as a delivery system for other health-promoting ingredients, such as plant sterols and stanols, remains relatively underexplored.

Plant sterols and stanols (collectively known as phytosterols) are naturally occurring compounds found in small quantities in various plant-based foods, including vegetable oils, nuts, seeds, grains, and legumes [4]. Due to their structural similarity to cholesterol, these compounds compete with cholesterol for absorption in the intestines, leading to a reduction in total cholesterol levels, particularly low-density lipoprotein (LDL) cholesterol [5]. The cholesterol-lowering effects of plant sterols and stanols are well established, with numerous studies confirming their efficacy. For example, Katan et al. [6] demonstrated that the daily intake of 2–3 g of plant sterols or stanols can lower LDL cholesterol levels by 10–15%. This reduction is particularly noteworthy given the well-established link between elevated LDL cholesterol and an increased risk of cardiovascular diseases, such as atherosclerosis, myocardial infarction, and stroke [7].

The incorporation of plant sterols and stanols into various food products has been a significant focus of recent research and development, leading to the availability of a wide range of sterol-enriched foods on the market. Margarines and fat spreads are among the most common vehicles for delivering plant sterols and stanols, with brands such as Optima Cardio, Benecol, and Flora ProActiv being well-known examples [8,9]. These products have been demonstrated to effectively lower LDL cholesterol when consumed regularly [10]. Beyond fat spreads, dairy products such as milk, yogurt, and cheese have also been enriched with plant sterols, providing consumers with additional options for incorporating these cholesterol-lowering compounds into their diets [11]. Studies indicate that these enriched dairy products not only reduce cholesterol levels effectively but are also well accepted by consumers, with no significant impact on taste or texture [6].

In addition to dairy products and spreads, plant sterols and stanols have been incorporated into a variety of other food products, including breakfast cereals, snack bars, and beverages [12]. For instance, studies have shown that sterol-enriched orange juice and granola bars can contribute to a significant reduction in LDL cholesterol levels, offering convenient and versatile options for cholesterol management [13,14]. Furthermore, research by Jones et al. [13,15] has demonstrated that the consumption of plant sterol-enriched foods not only reduces cholesterol levels but also improves overall lipid profiles, providing a comprehensive approach to cardiovascular health. Importantly, previous research by Plat and Mensink [16] has highlighted that plant sterol-enriched foods are well tolerated and do not adversely affect the sensory qualities of the food matrix. Similarly, a study by AbuMweis and Jones [17] showed that the addition of plant sterols to various food matrices maintained consumer acceptability while delivering significant cholesterol-lowering benefits. However, some studies have reported that high-temperature processing and extended storage can impact the chemical and physical properties of phytosterol-enriched products due to the oxidation susceptibility of these unsaturated lipid compounds [18,19].

While dairy and cereal products have successfully delivered health benefits, the integration of plant sterols and stanols into confectionery products, particularly dark chocolate, represents a novel approach with significant potential. The use of sterol-enriched dark chocolate as a dietary supplement for cholesterol management has been highlighted by Demonty et al. [20], who noted that regular consumption of such products could play a crucial role in public health strategies aimed at reducing the prevalence of cardiovascular disease. Moreover, the antioxidant properties of dark chocolate, attributed to its high polyphenol content, may be further enhanced by the presence of plant sterols, which have also been shown to exhibit antioxidant activity [21]. Additionally, the fatty acid composition and phenolic compounds inherent in the matrix of dark chocolate may provide natural protection against phytosterol oxidation during the enriched product storage [22]. It is worth noting that chocolate’s lipophilic properties make it a promising vehicle for phytosterol supplementation. Efraim et al. [23] demonstrated that the incorporation of oil-based soy phytosterols did not affect the rheological properties of the chocolate, and sensory evaluations indicated that phytosterol-enriched chocolate yielded results comparable to those of standard chocolate.

This study aims to expand the existing body of knowledge by examining the effects of enriching dark chocolate with plant sterols and stanols, specifically using preparation composed of phytosterol esters. Sterol and stanol contents were analyzed chromatographically, while antioxidant activity was assessed via the DPPH assay, and total phenolic content (TPC) was determined spectrophotometrically using the Folin–Ciocalteu method. Additionally, sensory evaluation was a key component of this study, as the acceptability of functional foods is strongly influenced by taste and texture. Previous studies, such as those by Gylling and Simonen [24], have underscored the importance of maintaining the sensory quality of plant sterol-enriched foods to ensure consumer compliance and product success.

## 2. Materials and Methods

### 2.1. Chemicals

Trolox (6-hydroxy-2,5,7,8-tetramethylchromane-2-carboxylic acid), DPPH (2,2-diphenyl-1-picrylhydrazyl) radical, gallic acid, 5α-cholestane, and HPLC-grade solvents and reagents such as acetonitrile, ammonium formate, formic acid, *n*-hexane, methyl tert-butyl ether (MTBE), and Sylon BTZ reagent were purchased from Sigma Aldrich (Poznań, Poland). Analytical-grade reagents such as acetic acid, acetone, anhydrous pyridine, diethyl ether, Folin–Ciocalteu (F-C) reagent, methanol, potassium hydroxide, sodium carbonate, sodium hydroxide, and sodium sulfate were supplied by Chempur (Piekary Śląskie, Poland).

### 2.2. Materials

The materials used in this study to prepare dark chocolate samples enriched with plant sterols included a carefully selected base composition and functional ingredients to ensure both high-quality sensory properties and the incorporation of health-promoting compounds. The base chocolate mass (purchased from Barry Callebaut Manufacturing, Łódź, Poland) consisted of cocoa mass, sugar, soy lecithin (as an emulsifier), and natural vanilla flavoring. The dry matter content of the chocolate mass was standardized to 68% cocoa solids, of which 31.7% were fat-free cocoa solids. The raw material was chosen to deliver a premium chocolate with desirable texture and flavor across all experimental samples.

For phytosterol enrichment, Vegapure^®^ 95 WE was used, which is a phytosterol ester preparation obtained through the esterification of free plant sterols with fatty acids derived from soybean oil. This functional ingredient was supplied by BASF SE (Ludwigshafen am Rhein, Germany), a recognized leader in the production of bioactive and functional food ingredients. The composition (% of total area in gas chromatography analysis) of the phytosterol preparation was approximately 69.1% sitosterol, 7.9% sitostanol, 15.3% campesterol, 1.0% campestanol, 0.8% stigmasterol, 2.8% brassicasterol, and 2.7% other sterols and stanols. 

### 2.3. Preparation of Chocolate Samples

Chocolate samples were produced under industrial conditions at Confectionery Factory Kopernik S.A. (Toruń, Poland). The phytosterol enrichment process was meticulously executed to ensure consistent quality across all samples. The active ingredient, Vegapure^®^ 95 WE (phytosterol esters), was incorporated into the chocolate mass through two distinct methods to assess the impact of timing on the final product characteristics. In the first method, the phytosterols were introduced during the conching process (CP), a phase in which the chocolate mass is refined to enhance its flavor and texture. In the second method, the phytosterols were added during the tempering process (TP), directly to the tempered chocolate prior to molding.

The production process began by melting the chocolate mass using a Pomati T-Line tempering machine (Codogno LO, Italy), which precisely controlled the temperature cycles necessary for proper cocoa butter crystallization, a crucial factor in achieving the desired texture and appearance of the chocolate. Following tempering, the chocolate was molded with careful attention to ensure accurate mold filling, while controlling the cooling and crystallization stages. This equipment ensured homogeneous distribution of the sterols within the chocolate matrix, preventing any separation or uneven mixing. The various concentrations (3, 6, 9, and 12%) of Vegapure^®^ 95 WE were incorporated into the liquid chocolate, followed by a 5 h homogenization period in the tank (simulating CP) or 10 min of mixing prior to pouring into the molds (simulating TP). Each batch of chocolate was produced in 50 kg quantities. Following production, the chocolate was packaged in polyethylene bags with zip closures and stored for 24 h in a dry place at a temperature below 20 °C prior to analysis.

The experimental design involved preparing nine different chocolate samples, as detailed in Table 1. These sample variations were developed to investigate how different production methods and the timing of phytosterol incorporation affect the sensory and nutritional quality of the final product.

### 2.4. Determination of Phytosterol Content

Sterol content was determined according to the AOCS Official Method Ch 6-91 [25]. For each analysis, 50 mg of fat was subjected to saponification with 1M KOH in methanol for 18 h at room temperature. The sterol fraction was then extracted three times using a solvent mixture of *n*-hexane and methyl tert-butyl ether (MTBE) in a 1:1 (*v*/*v*) ratio. After solvent evaporation under a nitrogen stream, the residue was dissolved in anhydrous pyridine and silylated using Sylon BTZ reagent. The resulting silylated derivatives were analyzed via gas chromatography (GC) on an Agilent Technologies 6890 system equipped with a DB-35MS capillary column (25 m × 0.20 mm × 0.33 μm) and a flame ionization detector (FID). A sample volume of 1 μL was injected in splitless mode using an automatic injector. Sterol separation was performed in a temperature-programmed oven, starting at 100 °C (held for 5 min), then ramped to 250 °C at 25 °C/min, and further increased to 290 °C at 3 °C/min. The final temperature of 290 °C was held for 20 min. Both the detector and injection port temperatures were maintained at 300 °C. Hydrogen served as the carrier gas at a flow rate of 1.5 mL/min. 5α-Cholestane was used as the internal standard, and sterols were identified by comparing their retention times with those of standard compounds.

### 2.5. Extraction of Antioxidants

The extraction procedure was as follows [26]: Approximately 2 g of each finely ground chocolate sample were weighed using a Radwag AS 220/C2 analytical balance (Poznań, Poland). The weighed samples were then quantitatively transferred into 100 mL glass-stoppered flasks. The extraction was performed using a mixture of three solvents: acetone (70%), distilled water (29.8%), and acetic acid (0.2%). The samples were mechanically shaken on a Chemland SK-O-330-PRO (Stargard, Poland) shaker for 30 min to ensure thorough extraction. After the extraction, the samples were filtered. The extraction procedure was repeated twice to ensure maximum recovery of phenolic compounds. The combined extracts, totaling 20 mL, were pooled together and stored in a refrigerator until further analysis for DPPH antioxidant capacity (AC) and total phenolic content (TPC) could be performed.

### 2.6. Determination of Total Phenolic Content

Total phenolic content was determined using the Folin–Ciocalteu colorimetric method [27]. Briefly, 0.05 mL of chocolate extract was transferred into 10 mL calibration flask, 0.5 mL of Folin–Ciocalteu reagent added and shaken (3 min). Next, 1 mL of saturated sodium carbonate solution was added and made up to the mark with redistilled water. After 45 min, the solution was centrifuged at 10,000 rpm (15 min) and absorbance at 725 nm was measured against a reagent blank. Three calibration curves were prepared for the gallic acid working solutions in the concentration range of 0.035–10.51 μg/mL. The least squares method was applied to calculate the line equation y = (0.1526 ± 0.00062)x + (0.0541 ± 0.0141), which presents a determination coefficient (R^2^) of 0.9974. The TPC was expressed as mg of gallic acid equivalents (GAE) per 100 g of chocolate sample.

### 2.7. Determination of Antioxidant Properties

The AC was evaluated using the DPPH assay, following the methodology developed originally by Brand-Williams et al. [28] with some modifications. In brief, 0.5 mL of chocolate extract (or Trolox standard solutions) was added to 1.5 mL of methanol and 0.5 mL of DPPH methanolic solution. The mixture was shaken vigorously and left in darkness for 15 min. The absorbance was measured at 517 nm against a reagent blank (2 mL of methanol + 0.5 mL of DPPH methanolic solution (c = 304 μmol/L) using a Spectrophotometer UV-Vis Hitachi U-2900 (Tokyo, Japan) in a 1 cm quartz cell. However, DPPH values expressed as micromoles of Trolox equivalents (TE) per 100 g of chocolate samples were obtained from the following linear relationship: f(concentration of Trolox) = % DPPH scavenging for Trolox standard solutions. Calibration curves were prepared using working Trolox solutions in methanol between 0.01 and 0.1 μmol/mL. Three calibration curves were plotted using the least squares method resulting in the equation y = (624.30 ± 2.57)x + (4.36 ± 0.14) and determination coefficient R^2^ = 0.9991.

### 2.8. Sensory Acceptance Test

A sensory evaluation of the enriched chocolates was conducted using an acceptance test with 86 untrained panelists (40 males and 46 females), aged 18 to 63, all of whom were regular chocolate consumers. The evaluation took place two weeks after the samples were prepared, employing a 9-point hedonic scale ranging from 1 (disliked extremely) to 9 (liked extremely), in accordance with the methodology outlined by Wichchukit and O’Mahony [29]. Panelists were asked to assess various sensory attributes of the chocolate, including appearance (color and glossiness), aroma, initial bite (crunch), cocoa flavor intensity, bitterness, sweetness, acidity, smoothness (texture/mouthfeel), melting behavior, aftertaste (one-minute post-consumption), and overall liking.

Three different chocolate samples were evaluated: Ch-CS, Ch3%S-TP, and Ch12%S-TP. The samples were presented in odor-free plastic containers with lids, each marked with a unique three-digit code in a randomized order to minimize order bias. Warm dark tea was provided to the panelists for palate cleansing between samples, ensuring precise and unbiased sensory assessment of each chocolate.

Panelists voluntarily participated in this study and, before sensory evaluation, read and signed the free and informed consent form. The sensory analysis was conducted in the sensory laboratory, where individual special cabinets equipped with controlled lighting were provided for each panelist.

### 2.9. Data Analysis

The DPPH and TPC measurements were conducted in five repetitions, while the results of phytosterols were taken in triplicate and presented as the average of two significantly similar determinations. All measurements were reported as means ± standard deviation (SD). All chemical analyses were analyzed using Statistica 13.1 PL software (StatSoft, Kraków, Poland) at a significance level of *p* ≤ 0.05. The differences between samples were determined using a one-way ANOVA with a Tukey’s test.

## 3. Results

### 3.1. Phytosterol Content in Dark Chocolates

**Figure 1:**
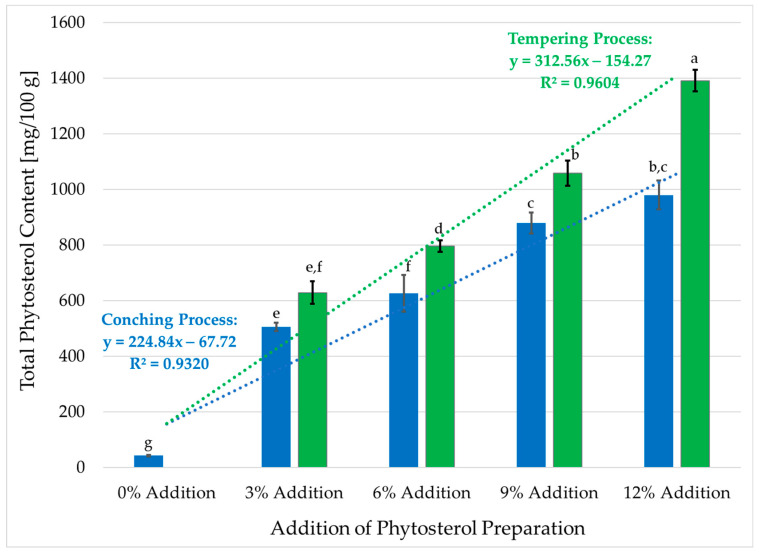
Total sterols content in chocolate samples with different addition of phytosterol esters; *n* = 2; $\bar{x}$ ± SD—mean value *±* standard deviation; different letters (a–g) indicate significant differences (one-way ANOVA and Tukey’s test, *p* ≤ 0.05).

The sterol and stanol composition of dark chocolate samples enriched with varying concentrations of phytosterol preparation are presented in Table 2, while the total phytosterol content of the samples is shown in Figure 1.

The analysis revealed that 100 g of the control sample (without phytosterol preparation) contained a total of 42.70 mg of phytosterols (Figure 1), primarily composed of stigmasterol (10.76 mg/100 g) and sitosterol (24.28 mg/100 g), with smaller amounts of cholesterol (0.79 mg/100 g), campesterol (4.42 mg/100 g), Δ5-avenasterol (1.25 mg/100 g), and cycloartenol (1.20 mg/100 g).

The addition of phytosterol preparation resulted in a significant increase in sterol content across all enriched chocolate samples. In chocolate containing 3% phytosterol preparation, the total sterol content rose to 505.28 mg/100 g when incorporated during the conching process (CP) and 629.11 mg/100 g when incorporated during the tempering process (TP). This increase was primarily driven by a substantial rise in sitosterol (388.85–485.45 mg/100 g), campesterol (44.92–49.07 mg/100 g), and sitostanol (39.09–55.76 mg/100 g). Additionally, stigmasterol (9.57–13.34 mg/100 g), campestanol (6.44–10.55 mg/100 g), brassicasterol (5.10–8.25 mg/100 g), as well as Δ5-avenasterol, cycloartenol, and Δ7-avenasterol (2–4 mg/100 g), showed elevated concentrations compared to the control sample. As the concentration of phytosterol preparation in the chocolate increased, the total sterol content demonstrated a proportional rise. Regression equations determined (Figure 1) indicated that a one-percent increase in the addition of Vegapure^®^ 95 WE corresponded to a 75% increase in total phytosterol content for TP and a 144% increase for CP.

The highest sterol content was observed in chocolate enriched with 12% phytosterol preparation during the tempering process (TP), where the total phytosterol concentration reached 1390.94 mg/100 g (Figure 1), representing a more than 30-fold increase compared to the control sample. In this enriched chocolate, sitosterol was the predominant sterol (1117.68 mg/100 g), followed by campesterol (119.10 mg/100 g), sitostanol (76.42 mg/100 g), and campestanol (24.14 mg/100 g). The concentrations of other phytosterols in the sample were low, not exceeding 18 mg/100 g. Notably, cholesterol levels did not follow a consistent trend with increasing phytosterol concentrations, fluctuating between 0.58 and 1.05 mg/100 g across all samples (Table 1).

These findings clearly demonstrate the efficacy of phytosterol preparation in significantly enhancing the plant sterol content of dark chocolate. The substantial increase in sitosterol, alongside the presence of other sterols such as campesterol and stigmasterol, underscores the potential of sterol-enriched chocolate as a functional food designed to support cardiovascular health through cholesterol management. The proportional increase in sterol content with higher phytosterol concentrations indicates a direct relationship between the amount of added sterols and the final sterol profile of the chocolate, which is essential for maximizing the health benefits of such functional foods.

### 3.2. Antioxidant Properties of Dark Chocolates

The antioxidant capacity (AC) and total phenolic content (TPC) of dark chocolate, with and without sterol enrichment during the conching and tempering processes, are presented in Table 3. 

Sterol esters were incorporated at concentrations ranging from 3% to 12%, regardless of the stage at which they were added. The AC and TPC data suggest that the addition of sterol esters to dark chocolate post-tempering results in enhancements in both antioxidant capacity and total phenolic content. Notably, the chocolate enriched with 3% sterols added during the tempering process (Ch3%S-TP) exhibited approximately double the AC and TPC values compared to the control sample (Ch-CS). Further increases in phytosterol preparation concentrations (6%, 9%, and 12%) added during the tempering process led to a progressive rise in AC and TPC values compared to Ch-CS. Interestingly, the chocolate containing 6% phytosterols added displayed a slight improvement in antioxidant potential over unenriched chocolate. This phenomenon warrants a more detailed investigation of specific antioxidant compounds and oxidation products present in dark chocolate to ascertain whether the 6% phytosterol addition triggered an interaction with oxidation products, potentially leading to the degradation of antioxidant constituents. Increasing the dose of phytosterols introduced during tempering resulted in a 19% and 13% increase in AC for Ch9%S-TP and Ch12%S-TP, respectively. In parallel, the phenolic content rose by 34% and 9% compared to the 6% sterol-enriched sample (Ch6%S-TP). Although dark chocolate enriched with the highest phytosterol preparation concentration during tempering process (Ch12%S-TP) exhibited an antioxidant potential (measured by DPPH and F-C assays) approximately 25% lower than the sample with 3% phytosterol preparation (Ch3%S-TP), it still demonstrated significantly higher values—by approximately 47% (AC) and 74% (TPC)—than the control sample (Ch-CS).

Conversely, when phytosterol esters were incorporated before tempering (during conching process), increasing their concentration from 3% to 12% led to a decline in antioxidant properties, as determined by the DPPH and FC assays. Dark chocolates enriched with 9% and 12% phytosterols added during the conching process displayed antioxidant activity comparable to chocolate without phytosterol preparation. Furthermore, dark chocolate containing the highest concentration of phytosterol preparation introduced during the conching process exhibited lower TPC than unenriched chocolate. This observation suggests that the conching process temperature (50 °C) may promote the formation of oxidation products from both the added sterols and inherent antioxidant components, leading to the degradation of antioxidant compounds in the evaluated chocolates. These findings emphasize the influence of the timing and concentration of phytosterol on the antioxidant properties of enriched dark chocolate.

### 3.3. Sensory Acceptance of Dark Chocolates

The results of the sensory evaluation (Table 4) indicated that the enrichment of chocolate with phytosterol esters at varying concentrations influenced several sensory attributes in distinct ways.

In terms of visual assessment, the chocolate containing 12% phytosterol preparation received the highest rating for color (8.62), indicating that higher sterol content may enhance the visual appeal of the product. However, glossiness was rated highest in the control sample (8.84), with a slight reduction observed in both the 3% and 12% phytosterol-enriched samples (8.20). This suggests that while phytosterol enrichment may improve color, it may also slightly diminish perceived gloss, a key factor in consumer perception of premium chocolate.

The aroma of the chocolate samples was positively influenced by the addition of phytosterols, with the 12% phytosterol sample achieving the highest score (8.47), indicating that higher phytosterol concentrations may enhance the aromatic profile of the chocolate. In contrast, the initial bite, or “crunch,” was negatively impacted by phytosterol preparation addition. The control sample scored the highest (7.62), while the 12% sample showed a significantly lower score (6.37), indicating that higher levels of phytosterol preparation may adversely affect texture, particularly in terms of the crispness experienced during the initial bite.

Regarding cocoa flavor intensity, the 3% phytosterol-enriched sample received the highest rating (7.84), followed closely by the 12% sample (7.64) and the control sample (7.55). This suggests that moderate levels of sterols may enhance the perception of cocoa flavor. Bitterness showed a slight increase with the addition of phytosterol preparation, with the 3% phytosterol sample receiving the highest score (7.72). However, the differences in bitterness between samples were minimal, suggesting that the influence of sterols on bitterness is modest and could be advantageous in dark chocolate formulations. Sweetness ratings were similarly close among samples, with the 3% sample receiving the highest score (7.71), while the 12% sample scored slightly lower (7.26). This implies that higher phytosterol concentrations may slightly reduce perceived sweetness, potentially affecting the overall flavor profile.

Acidity was most pronounced in the 12% sample (7.56), indicating that higher phytosterol concentrations may amplify acidic notes, which could alter the chocolate’s overall sensory profile. In terms of smoothness (texture/mouthfeel), the 12% phytosterol-enriched chocolate scored highest (8.17), suggesting that the addition of sterols enhances smoothness, possibly due to the increased fat content from the phytosterol oil mass. Similarly, melting behavior was rated highest for the 12% sample (7.80), suggesting that higher phytosterol concentrations improve melting properties, leading to a more pleasant mouthfeel as the chocolate melts. However, the aftertaste of the 12% sample was perceived less favorably (6.95) compared to the control (8.37), suggesting that higher levels of phytosterol preparation may introduce an undesirable aftertaste, potentially detracting from the overall sensory experience.

Overall liking was highest for the control sample (7.74), with slight decreases observed in the 3% (7.64) and 12% (7.50) samples. This suggests that while moderate phytosterol preparation enrichment is generally well received, higher concentrations may reduce overall consumer satisfaction.

In summary, the incorporation of phytosterols had both positive and negative effects on the sensory attributes of chocolate. The moderate level of phytosterol preparation (3%) appeared to enhance certain attributes, such as cocoa flavor intensity and smoothness, without significantly diminishing overall liking. However, higher levels of enrichment (12%) negatively affected texture (crispness), aftertaste, and overall consumer satisfaction. These findings highlight the importance of optimizing phytosterol concentrations in fortified chocolate to preserve sensory quality and consumer appeal, ensuring that the functional benefits of phytosterols do not compromise the overall sensory enjoyment of the product.

## 4. Discussion

This study on the enriching of dark chocolate with a plant sterol preparation provides comprehensive insights into the effects of varying phytosterol concentrations on both the chemical and sensory properties of chocolate. The enrichment resulted in a significant increase in total phytosterol content, with sitosterol being the most abundant compound, particularly in samples with higher concentrations of phytosterols. The findings indicate that the addition of sterols during the tempering stage enhanced both antioxidant capacity and total phenolic content, as measured by DPPH radical scavenging activity and TPC values. These results are consistent with the work of Tolve et al. [30], who reported positive effects of phytosterol addition on the antioxidant properties of chocolate.

Conversely, the incorporation of phytosterols during the conching stage led to a reduction in antioxidant properties at higher concentrations, likely due to heat-induced degradation of sterols and phenolic compounds. This finding aligns with the conclusions of Bodbodak et al. [31], who noted that thermal processing could negatively impact the stability of antioxidants in enriched foods.

Regarding sensory evaluation, this study revealed that a moderate level of phytosterol preparation (3%) improved sensory attributes such as aroma, cocoa flavor intensity, bitterness, sweetness, smoothness, and melting behavior. This finding is consistent with data reported by Faccinetto-Beltrán et al. [32], which emphasizes that moderate additions of bioactive ingredients in chocolate enhanced sensory acceptance. In contrast, higher concentrations (12%) adversely affected texture, aftertaste, and overall liking, diminishing the initial crunch and increasing acidity. This observation is in line with the work of Urbańska et al. [33], who found that excessive additions of bioactive compounds, including polyphenols, could detract from chocolate’s flavor profile and reduce consumer acceptance. This study underscores the necessity of balancing functional enrichment with sensory qualities, a common challenge in functional food research.

In comparison, studies on other enriched foods, such as plant sterol-enriched yogurts, have demonstrated that even higher phytosterol concentrations did not significantly impact sensory properties, as noted by Izadi et al. [34]. This contrast suggests that the food matrix plays a critical role in how enrichment affects texture and flavor, with solid products like chocolate being more sensitive to changes in mouthfeel and aftertaste compared to semi-liquid products such as yogurt.

## 5. Conclusions

This study concludes that while the addition of phytosterol preparation significantly enhances the health benefits of chocolate by increasing sterol content and antioxidant capacity, careful consideration must be given to the concentration and timing of enrichment. Moderate levels of enrichment offer the optimal balance between functional benefits and sensory acceptability, aligning with findings from broader research on functional foods. Future research could explore alternative methods for phytosterol incorporation or the use of stabilizing ingredients to mitigate the adverse sensory effects observed at higher levels of fortification.

In summary, enriching dark chocolate with plant sterols represents an innovative approach to developing functional foods that offer dual benefits: enhanced antioxidant properties and cholesterol-lowering effects. By combining the health-promoting attributes of dark chocolate with those of plant sterols, this study aims to contribute to the growing field of functional foods and provide insights into the development of novel products that support cardiovascular health.

## Figures and Tables

**Table 1:** Prepared chocolate samples.

Sample Code	Addition of Phytosterol Preparation	Processing Stage of Phytosterol Preparation Incorporation	Photo
Ch-CS (control sample)	0%	-	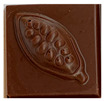
Ch3%S-CP	3%	Conching process	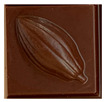
Ch6%S-CP	6%	Conching process	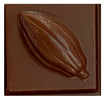
Ch9%S-CP	9%	Conching process	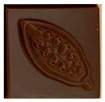
Ch12%S-CP	12%	Conching process	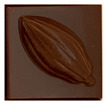
Ch3%S-TP	3%	Tempering process	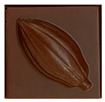
Ch6%S-TP	6%	Tempering process	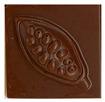
Ch9%S-TP	9%	Tempering process	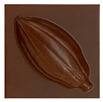
Ch12%S-TP	12%	Tempering process	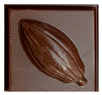

**Table 2:** Contents (mg/100 g) of sterols and stanols in chocolate samples.

Chocolate	Cholesterol	Brassicasterol	Campesterol	Campestanol	Stigmasterol	Sitosterol	Sitostanol	Δ5-Avenasterol	Cycloartenol	Δ7-Avenasterol
Ch-CS	0.79 ± 0.12 ^a^	0.00 ± 0.00 ^f^	4.42 ± 0.60 ^e^	0.00 ± 0.00 ^d^	10.76 ± 0.69 ^b^	24.28 ± 1.43 ^f^	0.00 ± 0.00 ^b^	1.25 ± 0.16 ^e^	1.20 ± 0.43 ^d^	0.00 ± 0.00 ^c^
Ch3%S-CP	0.88 ± 0.42 ^a^	8.25 ± 0.30 ^d,e^	44.92 ± 1.78 ^d^	6.44 ± 0.88 ^c,d^	9.57 ± 0.13 ^b^	388.85 ± 13.43 ^e^	39.09 ± 6.05 ^a,b^	3.14 ± 0.16 ^d,e^	2.40 ± 0.20 ^c,d^	1.74 ± 0.07 ^b,c^
Ch6%S-CP	0.75 ± 0.13 ^a^	5.16 ± 1.05 ^e^	52.99 ± 7.08 ^c,d^	10.59 ± 0.46 ^b,c^	13.70 ± 1.32 ^a,b^	479.12 ± 55.30 ^e^	55.91 ± 2.51 ^a^	3.57 ± 0.82 ^c,d,e^	2.46 ± 0.54 ^b,c,d^	2.36 ± 0.76 ^a,b,c^
Ch9%S-CP	0.58 ± 0.30 ^a^	9.15 ± 1.12 ^c,d^	77.27 ± 4.71 ^b,c^	12.87 ± 1.11 ^b,c^	10.34 ± 0.46 ^b^	702.00 ± 31.20 ^c,d^	53.18 ± 15.03 ^a^	6.27 ± 0.25 ^a,b,c^	4.64 ± 0.80 ^a,b,c^	3.40 ± 0.28 ^a,b^
Ch12%S-CP	1.02 ± 0.15 ^a^	14.01 ± 1.43 ^a,b^	88.21 ± 14.90 ^b^	12.91 ± 2.07 ^b,c^	14.40 ± 1.11 ^a,b^	781.40 ± 35.30 ^b,c^	53.76 ± 15.60 ^a^	7.04 ± 1.02 ^a,b^	4.26 ± 0.72 ^a,b,c^	2.67 ± 0.78 ^a,b^
Ch3%S-TP	0.82 ±0.35 ^a^	5.10 ± 0.73 ^e^	49.07 ± 8.87 ^c,d^	10.55 ± 1.10 ^b,c^	13.34 ± 1.98 ^a,b^	485.45 ± 28.40 ^e^	55.76 ± 21.10 ^a^	4.02 ± 1.59 ^c,d,e^	2.90 ± 1.01 ^b,c,d^	2.10 ± 0.04 ^a,b,c^
Ch6%S-TP	1.05 ± 0.40 ^a^	8.77 ± 0.25 ^d^	69.75 ± 1.63 ^b,c,d^	12.00 ± 0.63 ^b,c^	13.70 ± 0.75 ^a,b^	615.75 ± 18.60 ^d^	63.75 ± 10.81 ^a^	4.24 ± 0.37 ^b,c,d^	4.04 ± 0.30 ^b,c^	3.01 ± 0.48 ^a,b^
Ch9%S-TP	0.67 ± 0.19 ^a^	12.08 ± 0.52 ^b,c^	98.06 ± 3.22 ^a,b^	15.38 ± 4.18 ^b^	12.89 ± 3.25 ^a,b^	836.63 ± 44.70 ^b^	67.54 ± 5.58 ^a^	7.10 ± 0.66 ^a,b^	4.73 ± 0.43 ^a,b^	3.11 ± 0.47 ^a,b^
Ch12%S-TP	0.77 ±0.31 ^a^	15.28 ± 0.57 ^a^	119.10 ± 10.24 ^a^	24.14 ± 3.63 ^a^	17.61 ± 0.91 ^a^	1117.68 ± 28.00 ^a^	76.42 ± 7.14 ^a^	8.96 ± 0.43 ^a^	6.53 ± 0.28 ^a^	4.45 ± 1.35 ^a^

*n* = 2; $\bar{x}$ ± SD—mean value ± standard deviation; different letters (a–f) within the same column indicate significant differences (one-way ANOVA and Tukey’s test, *p* ≤ 0.05).

**Table 3:** Antioxidant capacity (AC) and total phenolic content (TPC) in dark chocolate with varying phytosterol preparation added during the conching (CP) and tempering (TP) processes.

Chocolate Sample	AC ± SD [μmol TE/100 g]	TPC ± SD [mg GAE/100 g]
Ch-CS	3700.72 ± 180.55 ^d^	918.33 ± 29.30 ^f^
Ch3%S-CP	6851.71 ± 232.53 ^a^	2011.14 ± 35.44 ^b^
Ch6%S-CP	4785.30 ± 196.58 ^c^	1303.25 ± 26.67 ^e^
Ch9%S-CP	3376.29 ± 151.10 ^d^	954.98 ± 26.55 ^f^
Ch12%S-CP	3471.11 ± 162.19 ^d^	772.06 ± 24.63 ^g^
Ch3%S-TP	7223.22 ± 226.21 ^a^	2076.09 ± 18.73 ^a^
Ch6%S-TP	4055.67 ± 175.24 ^a^	1086.29 ± 41.45 ^a^
Ch9%S-TP	4820.91 ± 157.78 ^c^	1457.68 ± 53.38 ^d^
Ch12%S-TP	5441.60 ± 106.45 ^b^	1599.49 ± 14.94 ^c^

*n* = 5; $\bar{x}$ ± SD—mean value ± standard deviation; different letters (a–g) within the same column indicate significant differences (one-way ANOVA and Tukey’s test, *p* ≤ 0.05).

**Table 4:** Sensory evaluation of selected chocolate samples.

Sensory Attributes	Ch-CS	Ch3%S-TP	Ch12%S-TP
Appearance color	8.36 ± 0.82 ^a,b^	8.26 ± 0.87 ^b^	8.62 ± 0.64 ^a^
Appearance glossiness	8.84 ± 0.37 ^a^	8.20 ± 0.85 ^b^	8.20 ± 0.91 ^b^
Aroma	7.51 ± 0.84 ^c^	8.01 ± 0.82 ^b^	8.47 ± 0.73 ^a^
Initial bite	7.62 ± 0.80 ^a^	7.47 ± 0.97 ^a^	6.37 ± 0.99 ^b^
Cocoa flavor intensity	7.55 ± 0.73 ^a^	7.84 ± 0.70 ^a^	7.64 ± 1.31 ^a^
Bitterness	7.44 ± 0.81 ^a^	7.72 ± 0.75 ^a^	7.37 ± 1.32 ^a^
Sweetness	7.52 ± 0.79 ^a,b^	7.71 ± 0.81 ^a^	7.26 ± 1.60 ^b^
Acidity	7.40 ± 0.86 ^a^	7.31 ± 1.30 ^a^	7.56 ± 1.41 ^a^
Smoothness	7.70 ± 0.95 ^b^	7.84 ± 0.68 ^a,b^	8.17 ± 1.39 ^a^
Melting behavior	7.38 ± 1.06 ^a^	7.66 ± 0.70 ^a^	7.80 ± 1.70 ^a^
Aftertaste	8.37 ± 0.78 ^a^	8.14 ± 0.74 ^a^	6.95 ± 1.30 ^b^
Overall liking	7.74 ± 0.81 ^a^	7.64 ± 0.72 ^a^	7.50 ± 1.66 ^a^

*n* = 86; $\bar{x}$ ± SD—mean value ± standard deviation; different letters (a–c) within the same line indicate significant differences (one-way ANOVA and Tukey’s test, *p* ≤ 0.05).

## Data Availability

The original contributions presented in this study are included in the article; further inquiries can be directed to the corresponding author.
